# Graduate Study on the Continent.—Germany

**Published:** 1908-04-25

**Authors:** 


### HOSPITAL ADMINISTRATION.

### CONSTRUCTION AND ECONOMICS.

### GRADUATE STUDY ON THE CONTINENT.—GERMANY.

I.—GRADUATE STUDY IN GERMANY.

With the exception of America, there is probably no country in which graduate study is better organised than in Germany. Since the days of the Franco-German War, when special courses were instituted for civil surgeons desirous of helping their military colleagues, the movement to encourage such study has been gaining strength steadily. At first enthusiasm was confined to a small section, not of the leaders of the medical profession, but of energetic and far-sighted general practitioners. For—and the fact is worthy of notice—graduate study here owes its present possibilities largely to the energy of the "practising physician." Lately, however, he has been very materially helped by the leaders of the faculty. Their goodwill the movement has always had, but such things as have been effected were made possible not by kindly intentions merely, but by honest hard work, which has succeeded in putting graduate work so far as medicine is concerned, on a sure and highly satisfactory basis.

At the present moment the profession in Germany is hyperenthusiastic about graduate study. A large *Fortbildung's* association has been formed, with the Chancellor, Prince Von Bülow, as President, which has for its chief object the better organisation of post-graduate study. Centres are being established in every town of importance. The infirmaries, the military lazarets, and the special asylums, clinics and institutions, have cordially offered their support, and a splendid programme of vacation courses has been definitely arranged for the ensuing summer vacation. The general practitioner has been offered, through the energy of this important association, which should serve as a model for any post-graduate organisation, the fullest scope for improving his knowledge. The fees are in most cases merely nominal, in many cases the courses are quite free, and every inducement has been given the intending student to attach himself to one or other centre. With the Chancellor as President, the Association promises to be a powerful body, and as its main and, indeed, its only object is the furtherance and encouragement of graduate study, its future will be followed with interest by those who believe that the movement is destined to do great things.

So much for the German general practitioner. The *Ausländer* have a slightly more complicated route to follow, but even in their case every possible effort is made to guide them and to make the sign posts fully legible. The foreigner may either enter as a student or he may register as a "Praktische Arzt," or practising physician, for the purposes of participating in the benefits to be derived from the vacation courses.

1. To enter as a student the regulations are similar, in the main, at all the German Universities. Matriculation is an indispensable preliminary to studentship, and to be "inscribed" it is necessary that the applicant should show proof of having received such general education as may be deemed sufficient to excuse him from the matriculation examination demanded of all German students. Usually the matriculation certificate of an English or Colonial University is sufficient, provided it shows that the candidate has passed in Greek or Latin. Sometimes, as in Berlin, Halle, and some other Universities, an English matriculation certificate is not regarded as equivalent, and the candidate must possess a University degree. In addition, all applicants must produce a passport or credentials, and must show that they have "visible means of existence." The fee for matriculation varies from 18 marks (Berlin) to 8 marks (Tübingen), a reduction being made in cases where the applicant is already a matriculated student of some German University other than that which he wishes to join. Every student is required to attend at least one course of lectures in the faculty which he has joined during the *semester* (term). The terms for such courses vary. At Berlin, for instance, the fees are 120 marks for the whole term for all the lectures in surgery, including operations and demonstrations; at Erlangen they are much lower. Each student has to pay, at the beginning of the term, certain "extras," which are more or less the same at every University. At Berlin they are as follows: *Auditoriengeld*, 5 marks; subscription to the general students' fund, half a mark; subscription to the students' sick fund, 2 marks (this last ensures free medical attendance, nursing, and treatment in case of illness). In addition, medical students have to pay an "Institute fee" of 5 marks. The fees for the first half-year at the Berlin University for a graduate who wishes to study surgery, say, as a matriculated student will thus approximate 160-170 marks; further *semester* fees can be reckoned at 20 per cent. less. Special arrangements are made for the benefit of foreign graduates or diplomates who wish to obtain the degree of M.D. of a German University. Such candidates are usually required to attend at least one *semester* or term at the lectures of the University in the faculty they have chosen, and to lay before the censor an original work or thesis on some subject chosen by them. Such work must be original and show evidence of research; a mere enumeration of authorities and extracts will not suffice. If this thesis is approved of the candidate must pass an end examination in his special faculty—in medicine, a purely practical examination in all the various subjects ranging from bacteriology to medical zoology. The standard of this examination is a fairly high one, and a knowledge of German is, of course, absolutely essential.

Where the foreign graduate does not wish to stay any length of time at a University, but is yet desirous of benefiting by the regular lectures, he may, on application to the Rector of the University, obtain a card of entry as a *Gastzuhorer*, or honorary member. Such a "Hospitantenschein" is granted to women students as well as to men (it may be mentioned that no difference is made between the sexes at most Universities in the medical or philosophical faculties) on production by the applicant of the usual credentials and a written application setting forth the subjects in which the applicant is specially interested. At Berlin application should be made to the Secretary of the University, Room 8, University Buildings, Opera Place, any morning after ten o'clock. The applicant's diploma, without which a graduate student should not travel abroad, and passport, together with a fee of 12 marks, are all that are required. When the student leaves, his card of honorary membership should be returned to the Secretary. When the graduate has successfully passed these preliminaries he obtains the calendar (usually half a mark), which gives full particulars of all the faculties, names and addresses of the professors, times and subjects of lectures, and notes on special fees or courses. The *Verzeichnis der Vorlesungen* of the Berlin Friedrich-Wilhelm University for the summer term, commencing April 22 and ending August 15 next, gives, for example, particulars of 1,066 lecture-courses to be held during the term. Of these no fewer than 374 courses are given in the faculty of medicine and surgery.

The majority of visitors, however, will not be desirous of joining the University classes. For such vacation courses are given, with which I hope to deal fully in a future article. Suffice it for the present to state that for attendance at such courses the applicant has to show that he is a qualified practitioner, entitled, that is, to practise his profession in his own country. Vacation courses are held at all the German Universities, fees and requirements varying from time to time. Usually only German practitioners are admitted to such courses, but in many cases foreigners are allowed to join as honorary auditors.

For the man with plenty of time at his disposal the best way undoubtedly is to join the University. The fees are so low and the benefits so great that such a course is much to be preferred, where time permits of its being followed. By so doing the student is able to gain the utmost from his sojourn in the University town—to see German student life and to become acquainted with the novelties and the different methods of teaching and demonstration. Where such a course is impossible, the next best thing is to combine a University course, as a *Gastzuhorer*, with special lectures and, if possible, with a vacation course.

